# Pushing the Boundaries of Transcatheter Therapies

**DOI:** 10.1016/j.jaccas.2024.102750

**Published:** 2025-01-15

**Authors:** Giuliano Costa, Sofia Sammartino

**Affiliations:** Division of Cardiology, University Hospital Policlinico G. Rodolico-San Marco, University of Catania, Catania, Italy

**Keywords:** endovascular closure, pseudoaneurysm, tailored therapy, TAVR, transcatheter, valve-in-valve

**Figure:**
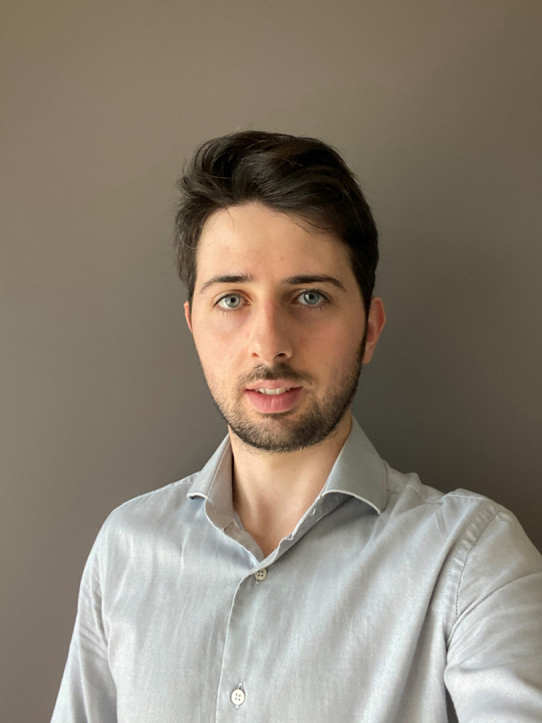

The Bentall procedure consists of replacement of the first portion of the aorta arising from the left ventricle with a valved conduit on which the coronary arteries are reimplanted. A rare complication of this procedure is the development of ascending aortic pseudoaneurysms (AAPs), most often located in the graft or coronary artery anastomoses. AAPs are generally asymptomatic and are detected during computed tomography (CT) follow-up. Conversely, they can cause symptoms secondary to compression of adjacent structures. Fatal AAP rupture can occur, as well as fistula formation resulting from erosion of surrounding tissues in response to growth of an AAP. Treatment of AAPs is recommended, with surgery the gold standard of intervention and percutaneous treatment an alternative, second-line approach.^1^

In this issue of *JACC: Case Reports,* Ali et al^2^ report the case of a 76-year-old male patient presenting with valve dysfunction after a previous Bentall procedure with a 27-mm BioIntegral BioConduit (BioIntegral Surgical). The patient presented with a dilated left ventricle with moderate systolic dysfunction and severe aortic regurgitation caused by prolapse of the right coronary cusp, but preprocedural CT assessment also revealed a large pseudoaneurysm (33 mm × 28 mm × 17mm) in the anterior wall of the ascending aorta that was eroding the sternum.

Given the patient’s high predicted surgical risk (European System for Cardiac Operative Risk Evaluation [EuroSCORE], 23%), which was increased by the location of the AAP behind the sternum, the heart team decided to proceed with transcatheter treatment for both the AAP and the valve dysfunction.

The endovascular treatment of AAP is particularly challenging because of the lack of ad hoc designed devices and the anatomical variability of these pseudoaneurysms.

This treatment may include the use of coil embolization or thrombin injections, or it may entail implantation of stent grafts, vascular plugs, or patent foramen ovale and left atrial appendage occluder devices.^3^, ^4^, ^5^, ^6^, ^7^ In their case report, Ali et al^2^ show a large AAP with a wide mouth. Vascular plugs or occluder devices were considered unsuitable both because of the angle between the AAP and the aorta and because of the location and dimensions of the AAP, with a high risk of incomplete AAP exclusion and dislocation and embolization of the devices.

The use of a stent graft for excluding an AAP requires adequate landing zones both proximally and distally, to ensure sparing of the coronary ostia at the proximal end and the origins of the supra-aortic trunks at the distal end.^8^

To fulfill these criteria and according to the ascending aorta dimensions in this patient, the operators used the proximal aortic extender cuff of the covered endograft Gore TAG Thoracic Branch Endoprosthesis (W.L. Gore & Associates) off-label for this purpose.

Because the planned procedure also included concomitant transcatheter aortic valve replacement (TAVR), the key procedural decision was whether AAP treatment would be carried out first or second.

Pure aortic regurgitation was a mechanism of valve failure in this patient; therefore, the operators decided to implant a 34-mm Evolut FX transcatheter heart valve (THV) (Medtronic), which, through its recapturability, helped the operators increase the safety and effectiveness of the procedure in this context. Nevertheless, the high stent frame of the valve was expected to interact with the implantation of the stent graft intended to be used for excluding the AAP.

Ali et al^2^ proceeded first with the treatment of AAP by implanting 2 overlapping, off-label stent-grafts. The subsequent TAVR procedure was facilitated by the anchoring mechanism guaranteed by the interaction of the THV outflow end with the aortic stent grafts.

This case highlights the role of a proper evaluation of preoperative CT analysis and emphasizes the importance of operators’ knowledge of devices in planning transcatheter solutions for complex scenarios. In particular, patients who have undergone previous cardiac surgery may have high-risk characteristics for redo surgery, and alternative approaches should be evaluated for the treatment of such patients, even those with complex, concomitant issues.

Tailored, patient-specific transcatheter strategies may drastically minimize the procedural risk and improve the management of such patients, but these strategies need to be implemented in high-expertise centers.

A trap can be hidden behind each step of such complex procedures, and bailout solutions need to be anticipated in the phase of procedural planning.

The future development of ad hoc, tailorable devices for treating AAP and TAVR for pure aortic regurgitation is desirable, to address the specific issues of transcatheter therapies for these 2 entities. Besides, the continuous development in delivery systems of transcatheter solutions will help operators to face complex anatomical scenarios.

The future of transcatheter solutions is going to expand more and more, with several benefits especially for complex patients. However, it must be kept in mind that safety has to match efficacy, and the key to success lies in the critical assessment of all potential solutions tailored case by case according to patients’ anatomy.

## Funding Support and Author Disclosures

The authors have reported that they have no relationships relevant to the contents of this paper to disclose.
